# A genome wide association study of pulmonary tuberculosis susceptibility in Indonesians

**DOI:** 10.1186/1471-2350-13-5

**Published:** 2012-01-13

**Authors:** Eileen Png, Bachti Alisjahbana, Edhyana Sahiratmadja, Sangkot Marzuki, Ron Nelwan, Yanina Balabanova, Vladyslav Nikolayevskyy, Francis Drobniewski, Sergey Nejentsev, Iskandar Adnan, Esther van de Vosse, Martin L Hibberd, Reinout van Crevel, Tom HM Ottenhoff, Mark Seielstad

**Affiliations:** 1 Human Genetics, Genome Institute of Singapore, 60 Biopolis Street, Singapore 138672; 2 Infectious Disease, Genome Institute of Singapore, 60 Biopolis Street, Singapore 138672; 3 Dept. of Internal Medicine, Faculty of Medicine Universitas Padjadjaran, Bandung, Indonesia; 4 Health Research Unit, Faculty of Medicine Universitas Padjadjaran, Bandung, Indonesia; 5 Dept. of Biochemistry, Faculty of Medicine Universitas Padjadjaran, Bandung, Indonesia; 6 Eijkman Institute for Molecular Biology, Jl. Diponegoro 69, Jakarta, Indonesia 10430; 7 Infectious Disease Working Group, Medical Faculty, University of Indonesia, Jakarta, Indonesia; 8 Samara Oblast Tuberculosis Dispensary, Samara City, Samara, Russian Federation; 9 Clinical TB and HIV Group and Health Protection Agency, National Mycobacterium Reference Laboratory, The Blizard Institute, Barts and the London School of Medicine, Queen Mary College, University of London, London, UK; 10 Department of Medicine, University of Cambridge, Cambridge, UK; 11 Dept of Infectious Diseases, Leiden University Medical Center, Albinusdreef 2, 2333 ZA Leiden, The Netherlands; 12 Department of Medicine, Radboud University Nijmegen Medical Centre, Nijmegen, The Netherlands; 13 Institute for Human Genetics, University of California, San Francisco, California 94143-0794, USA, and Blood Systems Research Institute, 270 Masonic Avenue, San Francisco, California 94118, USA

## Abstract

**Background:**

There is reason to expect strong genetic influences on the risk of developing active pulmonary tuberculosis (TB) among latently infected individuals. Many of the genome wide linkage and association studies (GWAS) to date have been conducted on African populations. In order to identify additional targets in genetically dissimilar populations, and to enhance our understanding of this disease, we performed a multi-stage GWAS in a Southeast Asian cohort from Indonesia.

**Methods:**

In stage 1, we used the Affymetrix 100 K SNP GeneChip marker set to genotype 259 Indonesian samples. After quality control filtering, 108 cases and 115 controls were analyzed for association of 95,207 SNPs. In stage 2, we attempted validation of 2,453 SNPs with promising associations from the first stage, in 1,189 individuals from the same Indonesian cohort, and finally in stage 3 we selected 251 SNPs from this stage to test TB association in an independent Caucasian cohort (n = 3,760) from Russia.

**Results:**

Our study suggests evidence of association (P = 0.0004-0.0067) for 8 independent loci (nominal significance P < 0.05), which are located within or near the following genes involved in immune signaling: *JAG1*, *DYNLRB2*, *EBF1*, *TMEFF2*, *CCL17*, *HAUS6*, *PENK* and *TXNDC4*.

**Conclusions:**

Mechanisms of immune defense suggested by some of the identified genes exhibit biological plausibility and may suggest novel pathways involved in the host containment of infection with TB.

## Background

Tuberculosis (TB) remains one of the leading causes of infection-associated mortality, with close to 10 million new cases and 2 million deaths annually [1,2]. Although *Mycobacterium tuberculosis* has infected around a third of the world's population, only 3-10% of those infected develop active disease during their lifetime [3]. More than 90% of infected individuals remain asymptomatic with a latent infection. This indicates that host immune/defense pathways are often highly effective in controlling this disease. Because the infection causes such a burden of disease in those unable to contain the infection, it is important to discover underlying mechanisms to aid the development of more effective interventions such as better vaccines and novel treatments for latent and active infection. Similarly, it is important to identify predictive biomarkers that might identify individuals who are most susceptible to developing active TB disease.

Studies of heritability using twins and other familial designs have convincingly implicated a genetic component contributing to outcomes of TB infection [4-7]. This has encouraged us to conduct a genome-wide search for genes relevant to pulmonary TB susceptibility and active disease. Although animal and other models of infection have implicated a small number of possible candidate genes, these often have ambiguous or disappointing patterns of replication in humans [8]. Furthermore, the testing of candidate gene hypotheses are severely limited by assumptions and limitations to our current knowledge of the relevant pathways of immune containment. A genome wide association study (GWAS), by contrast, can scan nearly the entire genome for variants associated with a phenotype, free from limiting hypotheses of biological plausibility. This innovation in the study of complex disease genetics in humans has proved successful in discovering novel genetic associations across a wide array of phenotypes and diseases [9,10]. In the case of TB, a GWAS on African populations has identified a susceptibility locus for TB at chromosome 18 q11.2 [10]. The variant implicated, rs4331426, lies within a gene-desert, with the risk allele relatively common in the African population studied, though it is found at much lower frequencies in other populations, making it difficult to replicate the reported association outside Africa [10].

In the current study, we embarked on a two-stage GWAS using the first generation Affymetrix 100 K SNP GeneChip marker set in an Indonesian population sample from Jakarta and Bandung, two cities on the island of Java (n = 1,448) [11]. In stage 1, we analyzed 95,207 SNPs of 108 cases and 115 controls, and synthesized 2,453 selected top SNPs (P < 0.05) on two Illumina GoldenGate customized arrays, for genotyping the remaining 1,189 independent Indonesian samples, as validation in the second stage. 251 promising SNPs (Indonesian 2 stages P < 0.05) from the initial Indonesian studies were subsequently selected for genotyping and testing TB association in an independent cohort from Russia (n = 3,760). We have detected several variants within or near genes involved in immunity, albeit with nominal significance. Nevertheless, the plausibility of biological mechanisms suggested by some of these immune genes encourages us to suggest these variants and genes for further study.

## Methods

### Subjects

#### Indonesian cohort

Indonesian TB patients and controls were enrolled from the cities of Jakarta and Bandung on the island of Java, Indonesia using a uniform enrollment protocol for all subjects [12]. 799 TB patients (mean age 32, range 14-75, 55.8% male, see Table 1) had been diagnosed by the local health care service using information about clinical symptoms, chest X-rays, and sputum smear. For all cases in this study, diagnosis was further confirmed by sputum culture of *M. tuberculosis*. Clinical information, as well as the patients' age, ethnicity, socio-economic status, and concurrent medical history were recorded in structured questionnaires. Patients with extra-pulmonary TB, diabetes mellitus (fasting blood glucose > 126 mg/dL), and HIV-positive subjects were excluded from the genetic study [13,14]. 746 sex- and age (+/- 10 year) matched control subjects from the same areas (mean age 33, range 15-70, 52.5% male), with no history of TB and showing no evidence of TB-related infiltrates in chest X-rays were enrolled from the same and neighboring households of the enrolled cases. First-degree related individuals among subjects were identified by genetics, and were excluded from further analysis.

**Table 1:** Demographic data of the study populations

	Indonesian cohort		Russian cohort	
	TB Patients (n = 799)	Controls (n = 746)	TB Patients (n = 1,912)	Controls (n = 2,104)
Age years (mean)	14-75 (32)	15-70 (33)	17-86 (44)	16-66 (30)
Gender male:female (%)	55.8% : 44.2%	52.5% : 47.5%	73.8% : 26.2%	75% : 25%
Self reported ethnicity (%)				
Caucasian	0	0	1912 (100%)	2104 (100%)
Javanese	675 (84.48%)	617 (82.71%)		
mixed (either parent Javanese)	26 (3.25%)	43 (5.76%)		
non-Javanese	59 (7.38%)	32 (4.29%)		
Unknown	39 (4.88%)	54 (7.24%)		

Self and parental ethnicities recorded during recruitment were used to characterize subjects with a Javanese origin from three groups -the Jawa, Betawi, and Sunda, which altogether comprised more than 80% of the total sample. Individuals in the non-Javanese category have both parents coming from other Indonesian Islands, whereas subjects with one parent from non-Javanese origin were considered having mixed parentage (Table 1). Population outliers were detected by genetics in stage 1 using the genome wide markers (n = 95,207 SNPs), and were excluded for further analysis. Subjects with self-reported ethnicity that were of non-Indonesian origin were excluded from stage 2 genotyping. This protocol was reviewed and approved by the relevant institutional review boards in Indonesia and the Netherlands.

#### Russian cohort

Russian TB patients and controls were collected at two cities, St. Petersburg (1,528 patients and 1,609 controls) and Samara (384 patients and 495 controls), using a uniform enrollment protocol for all samples, which has been described previously [15]. In summary (Table 1), 1,912 TB patients (mean age 43.8, range 17-86, 73.8% male) were confirmed as cases by sputum culture of *M. tuberculosis*. Patients with extra-pulmonary TB or HIV-positive were excluded from the genetic study. 2,104 (mean age 30, range 16-66, 75.0% male) local blood bank donors with no known history of TB were recruited as controls. Permissions were obtained from the local ethics committees in St. Petersburg and Samara, Russia, and Cambridge, UK, and had written informed consent from all participating subjects.

### Genotyping

#### Stage 1: GWAS in Indonesian cohort

For the initial genome-wide scan, 125 cases and 134 controls were genotyped for 116,204 SNPs with the Affymetrix 100 K Human mapping SNP set, according to the manufacturer's protocol. Genotype calling was performed using Affymetrix's BRLMM software [16].

For quality control purposes, subjects were excluded based on: call-rate <90% (n = 2), first-degree familial relationship (n = 7), discrepancies with reported gender (n = 4), population outliers in an analysis of the first two principal components (n = 4) (see Additional file 1, Supplementary Figure S1), and a diagnosis of diabetes mellitus (n = 19), which has been consistently identified as a risk factor for active TB disease. After sample exclusions, SNPs were filtered to remove those that were: non-autosomal (n = 2,355), unmapped in reference genome build 123 (n = 1,225), call-rate <90% (n = 402), minor allele frequency (MAF) < 0.01 (n = 16,905), and P-value of Hardy-Weinberg equilibrium (HWE) test (controls only) < 1 × 10^-7^ (n = 110). The resulting post-QC dataset of 108 cases and 115 controls analyzed for 95,207 SNPs was then utilized in the association study.

### Stage 2: validation in Indonesian cohort

Selected from the highest ranking SNP associations from stage 1, we synthesized 2,453 SNPs (P <0.05) on the Illumina GoldenGate customized array in two separate pools. As according to manufacturer's protocol, 1189 independent subjects (626 cases and 563 controls) from the same Indonesian study were genotyped on these GoldenGate arrays, and the BeadStudio GenCall software was used to call for genotype[17].

Quality control filtering was based on: sample call-rate <90% (n = 9), first degree familial relationship (n = 14), discrepancies with reported gender (n = 11), and history of diabetes mellitus (n = 15). Following sample exclusions, SNPs were filtered to remove those that are: unmapped in reference genome build 123 (n = 3), minor allele frequency (MAF) <0.01 (n = 44), and P-value for Hardy-Weinberg equilibrium (HWE) test (controls only) < 1 × 10-^7^ (n = 25). The resulting post-QC dataset of 600 cases and 540 controls genotyped for 2,381 SNPs was then utilized in the association analysis.

Assuming a multiplicative model, and a TB prevalence in Indonesia of 262 cases per 100,000 [1], the total sample size of the two stage Indonesian cohort has >80% power to detect associations for risk alleles ≥ 40% frequency, and OR ≥1.5, for an uncorrected significance threshold of *P* = 0.05, which is the nominal alpha we consider to suggest association [18]. However, to account for multiple testing a stringent Bonferroni corrected alpha of *P* = 5.25 × 10-^7^ (0.05/95,207) is required to declare genome wide significance in this study.

### Stage 3: testing TB association in Russian cohort

Among the top SNP associations detected in the first two stages involving Indonesian subjects, 251 promising SNPs (Indonesian 2 stages P < 0.05) were selected for synthesis in an oligo pool assay (OPA) of the GoldenGate assay, see Additional file 2, Supplementary Table S1. Genotyping of these SNPs was performed on 3,760 Russian subjects to test TB association in a large independent cohort. The BeadStudio GenCall software was used to call for genotype [17].

For quality control purpose, 144 subjects were excluded because of sample duplication, and discrepancies with reported gender. No other samples were excluded after filtering for call-rate <90%, or of having first-degree familial relationship. After sample exclusions, SNPs were filtered to remove those with minor allele frequency (MAF) <0.01 (n = 6), and P-value for Hardy-Weinberg equilibrium (HWE) tests (controls only) < 1 × 10-^7^ (n = 2). The resulting post-QC dataset of 1,837 cases and 1,779 controls genotyped for 243 SNPs was then utilized in the association analysis.

Assuming a multiplicative model, and a TB prevalence in Russia of 150 cases per 100,000 in the population[1], the overall sample size of the Russian cohort has at least 99% power to detect associations at risk allele ≥ 40% frequency and ORs ≥ 1.5, for an uncorrected significance threshold of *P* = 0.05, which is the nominal alpha we consider to suggest association [18].

### Analysis of population stratification

#### Indonesian cohort

As population stratification can confound case-control association studies [19-21], we performed a principal components (PC) analysis as implemented in EIGENSTRAT to identify and exclude 4 population outliers within PC1 and 2, from the Indonesian stage 1 dataset, see Additional file 1, Supplementary Figure S1[21]. The median chi-square statistics of the post quality controlled stage 1 genome wide loci yield a lambda inflation factor (Devlin and Roeder method) of only 1.003, which indicate that population stratification was minimal in this study to cause significant inflation to the test statistics, see Additional file 1, Supplementary Figure S2[19]. Hence, no further adjustments were made to correct the association tests for any inflation.

The marker density of stage 2 was insufficient for performing principal components analysis. Nevertheless, to avoid spurious genetic associations arising from population stratification, efforts were made to ensure subjects with self-reported ethnicity that were of non-Indonesian origin were excluded from genotyping. Furthermore, as described previously, to detect traces of population stratification in the Indonesian cohort, a large subset of individuals (330 cases and 368 controls) that are part of this study, were genotyped for an independent set of 299 ancestry informative markers. These SNPs were chosen to be more than 10 Kb away from any known gene, to have average minor allele frequencies around 30% and to be in linkage equilibrium with one another [22]. The result of the lambda inflation factor calculated according to the method of Devlin and Roeder [19], had a value close to 1, which further confirmed that there was minimal population stratification in this Indonesian cohort [22].

#### Russian cohort

In order to control for hidden population stratification due to potential admixture, all Russian subjects were genotyped for 15 ancestry-informative markers that was as reported previously [15]. We selected these markers among intergenic or intronic SNPs in the non-immune genes spread across the genome that have minor allele frequency of more than 10% in Europeans and over 65% difference in allele frequency between European- and Asian-derived populations [23]. As was reported previously, all ancestry-informative markers had similar allele frequency in TB patients and healthy subjects (chi-square test *P* > 0.13) thus, suggesting that major adjustments nor population stratification are likely in this sample [15].

### Analysis of relative detection

As cryptic relatedness among study subjects may artifactually inflate the statistics of association in case-control studies [24], the genotypes of markers that had undergone quality control (Stage 1 n = 95,207 SNPs, or Stage 2 n = 2,381 SNPs) were used in the Relpair software to find pairs of individuals who are more similar than expected by chance in a random sample [25]. Based on the calculated probabilities, we identified pairs with relationships of an extent expected for monozygous twins, full siblings, and parent-offspring. In each instance, the sample with the higher call-rate was retained in the analysis.

### Analysis of association statistics

After sample and SNP quality control, statistics of association were calculated using the PLINK software package [26]. For detecting associations in the first stage, Trend tests were performed on 108 cases and 115 controls with genotypes for 95,207 SNPs. Subsequently, for the combined association results over the entire Indonesian cohort, the Cochran-Mantel-Haenszel (CMH) test was used to perform a stratified analysis across the two stages for the 2,381 quality filtered SNPs that had been successfully genotyped in 708 cases and 655 controls. For the stage 3 sample from Russia, including enrollments from two cities, the CMH test was used to stratify the association analysis by city, and provide the test statistics after controlling for difference in sample location.

Finally, for the combined test statistics across all three stages of the analysis, the CMH test was performed to stratify the association analysis by cohort. A stringent Bonferroni corrected alpha of *P* = 5.25 × 10^-7^ (0.05/95,207) is required to declare genome wide significance in this study. However, due to sample size considerations in this study, we consider also associations with P-values as low as 0.05 to be suggestive of association.

## Results

The demographic characteristics of the participants of our study are displayed in Table 1. In this study, we tested SNPs across the genome for association with pulmonary TB, in three separate stages. First in the discovery phase of stage 1, following extensive quality control filtering on the data, we analyzed 95,207 SNPs in 108 cases and 115 controls from Indonesia for association with pulmonary TB (see Additional file 1, Supplementary Figures S2 and S3). Among the SNPs tested 4,719 SNPs exceed an uncorrected P < 0.05. The median chi-square of this study yields a genomic control inflation (λ_GC_) of only 1.003, to indicate that population stratification is minimal to cause significant inflation, hence further adjustments were not made to the test statistics.

In order to validate promising associations from the initial discovery phase, in the second stage, the validation phase, we analyzed 2,381 selected top SNPs (Stage 1 P < 0.05) in 708 cases and 655 controls from Indonesia. We identified 368 SNPs at this stage that were nominally significant (P < 0.05) in the combined stage analysis, suggesting association with pulmonary TB in the Indonesian population.

In order to study TB association in a large independent cohort, 243 of the above SNPs identified in Indonesia, were tested in stage 3 in 1,837 cases and 1,779 controls from Russia. In the combined meta-analysis, 9 SNPs (P = 0.0004-0.0067) were discovered to associate with pulmonary TB, independently across both Indonesian and Russian cohorts, albeit with nominal (P < 0.05) significance (see Table 2). These nine SNPs are located within or near the following genes: *JAG1, DYNLRB2, EBF1, TMEFF2, CCL17*, *HAUS6, PENK* and *TXNDC4.*

**Table 2:** Association results of nine significant SNPs from the combined meta-analysis of all three stages

SNP	Chr.	Gene		Risk allele	Stage 1 Indo. P*	OR	(95% CI)		Stage 2 Indo. P*	OR	(95% CI)		Indo. allele freq.	Stage 3 Russ. P*	OR	(95% CI)		Russ allele freq.	Indo. & Russ. P	OR	(95% CI)	
rs2273061	20	JAG1		G	0.004	1.80	1.18	2.72	0.01	1.24	1.05	1.46	0.28	0.008	1.14	1.03	1.25	0.43	0.0004	1.16	1.07	1.26
rs4461087	16	DYNLR		A	0.009	1.62	1.10	2.37	0.03	1.18	1.01	1.38	0.38	0.01	1.18	1.04	1.34	0.16	0.001	1.18	1.07	1.30
rs10515787	5	EBF1		A	0.006	0.57	0.38	0.88	0.02	0.81	0.68	0.96	0.26	0.02	0.73	0.56	0.96	0.03	0.001	0.79	0.68	0.91
rs10497744	2	TMEFF2	Both SNPs in LD r^2^ = 0.99 D' = 1.00	A	0.002	0.55	0.38	0.82	0.02	0.83	0.71	0.97	0.35	0.02	0.89	0.80	0.98	0.30	0.001	0.87	0.80	0.95
rs1020941	2	TMEFF2		C	0.004	0.57	0.38	0.83	0.03	0.84	0.72	0.98	0.35	0.03	0.89	0.81	0.99	0.30	0.002	0.88	0.81	0.95
rs188872	16	CCL17		A	0.004	0.51	0.33	0.78	0.02	0.82	0.70	0.97	0.30	0.04	0.89	0.80	0.99	0.25	0.002	0.87	0.80	0.95
rs10245298	7	HAUS6		A	0.03	2.37	1.09	5.16	0.03	1.40	1.04	1.89	0.07	0.04	1.18	1.01	1.39	0.09	0.005	1.23	1.06	1.41
rs6985962	8	PENK		C	0.02	2.01	1.12	3.61	0.04	1.26	1.01	1.59	0.13	0.047	1.14	1.00	1.29	0.15	0.006	1.17	1.05	1.31
rs1418267	9	TXNDC4		A	0.0004	3.19	1.71	5.99	0.04	1.28	1.01	1.62	0.12	0.04	1.11	1.01	1.22	0.40	0.007	1.13	1.03	1.23

Chr.- chromosome, LD- linkage disequilibrium, r^2^ - R square, D' - D prime, Indo.- Indonesia, P- P-value, OR- odds ratio, 95%

CI- 95% confidence interval, freq.- frequency, Russ.- Russia

• See Additional file 2, Supplementary Table S2 for genotype counts

## Discussion

Our TB association study extends across two genetically highly diverse populations. It combines GWAS in Indonesian population and follow-up genotyping of the best associated SNPs in a large independent cohort from Russia. Our data provide evidence of possible novel associations of genetic variants with pulmonary TB susceptibility. Among them (Table 2), one of our lowest *P* values is observed for rs2273061 (P 0.0004, O.R 1.16, 95%C.I. 1.07-1.26), which is in the transcript of JAG1. This protein is a ligand for the Notch receptor that plays a central part of the Notch signaling cascade [27]. Mouse macrophages infected with *M. bovis* BCG have been shown to up-regulate NOTCH1 signaling leading to SOCS3 expression via NOTCH1 mediated recruitment of NFκB and CSL to the SOCS3 promoter. As SOCS3 is a critical regulator of cytokine signaling, induction of this gene by mycobacteria could suggest a strategy to render infected macrophages unresponsive to interferon-gamma (IFN-γ), which is a central Th1 cytokine [28]. This modulation of host cell signaling response may be critical for a generalized suppression of inflammatory responses, and the persistence of mycobacteria within the host.

Notch signaling also plays a pivotal role in T cell lineage commitment, and another associated SNP, rs10515787 (P 0.0013, O.R. 0.79, 95%C.I. 0.68-0.91) is in the EBF1 gene, which is a central B cell lineage specification factor. In order for EBF1 to perform its role, it must partner with PAX5 through a feedback regulation to amplify B cell specific gene expression and solidify the commitment to the B cell pathway [29]. PAX5 is the guardian of B cell identity and functions by down regulating genes that are against B cell lineage, such as the M-CSF and NOTCH1, which are required for myeloid development and T cell lineage specification respectively [30,31]. This counteractive response of repressing NOTCH1 signaling that is not in favor of T cell promotion, might suggests an impact on the control of the intracellular infection of *M. tuberculosis*.

Two SNPs rs10497744 (P 0.0014, OR 0.87, 95%C.I. 0.80-0.95) and rs1020941 (P 0.0022, OR 0.88, 95%C.I. 0.81-0.95) in LD (r^*2*^ = 0.99, D' = 1.00) that are part of the associated list are near the TMEFF2 gene. This gene encodes a transmembrane protein with EGF (epidermal growth factor)-like and two follistatin-like domains 2, which is known to contribute to cell proliferation. Shedding of TMEFF2 from the ectodomain is a functionally important step to release the protein in its active form for inducing cellular proliferation. This functionally limiting step is highly mediated through an ADAM17 dependent autocrine fashion [32]. Incidentally, ADAM17 also has a prominent role in activating the cell-fate specification Notch signaling pathway, by controlling the shedding of Notch receptor and its ligand JAG1 [33], which is also our first target gene, mentioned above. An active ADAM17 regulates EGF receptor expression through activating NOTCH1 that was demonstrated to affect proliferation and survival of lung cancer cells, and tumorigenicity of non-small cell lung cancer [34]. However, on the other hand, inactivating NOTCH1 or ADAM17 resulted in substantial cell death, while EGFR inhibition predominantly induced cell arrest in lung cancer [34]. Studies have also shown ADAM17 actively mediates the shedding of pro-inflammatory factors in lung inflammation, and regulate immune cell recruitment and cytokines secretion that affects the physiology of this organ [35]. In pulmonary TB, the lung is the primary site of infection by *M. tuberculosis* where, responding to invasion, our body reacts by recruiting immune cells and pro-inflammatory cytokines to attack and control further dissemination of a pathogen by forming granuloma, which may also manifest in tissue damage.

Another example of biologically relevant candidate in our data is rs188872 (P 0.0023, O.R. 0.87, 95%C.I. 0.80-0.95), which is near the CCL17 cytokine gene. Lung granulomas in mice were reported to have enhanced CCL17 transcript levels after being stimulated with *M. bovis* antigen [36]. As a survival mechanism, pathogens such as *M. tuberculosis* are known to preferentially shift host cell response towards Th2 by instigating the production of Th2 cytokines. When in excess, it would consequently lead to immuno-suppression that might antagonize the Th1 mediated microbicidal actions. In natural infection *M. tuberculosis* may likely gain from this favorable condition to survive in infected patients.

Within the Indonesian study, the lowest P value is found in rs10497225 (P 1.52 × 10^-5^, O.R. 2.36, 95%C.I. 1.58-3.52), which is in the SLC4A10 gene, see Additional file 2, Supplementary Table S1. This solute carrier family 4, sodium bicarbonate transporter, member 10 (SLC4A10) gene is in a similar class of function as the ion transporter; SLC11A1 (alias NRAMP1), a well studied TB gene involved in iron metabolism and host resistance to pathogenic mycobacteria. Genetic variants of this gene have been associated with susceptibility to TB and leprosy[37,38]. However, we could not analyze rs10497225 in the Russian cohort because this SNP is rare (MAF 0.0007) in this population, and was excluded after failing MAF filter. In view of this, we believe some of the association signals could be affected by possible genetic differences between the host populations. As these SNPs are merely markers tagging the actual causal variants based on linkage disequilibrium (LD), differences in LD patterns and allele frequencies between differing ethnicities could affect the efficiency of transferring tags across populations and the power in detecting associations. This is notwithstanding the fact that the 100 K SNP GeneChip marker set used in Stage1 is a rather sparse collection of SNPs. The SNPs in this microarray capture (*r*^2^ ≥ 0.8) common variants in the Asian (JPT+CHB) and European (CEU) genomes at only 30% coverage[39], that are also undersampled in the coding regions, reducing the level of proxy to genes[40]. Hence, it is likely that certain regions in the genome are less adequately tagged with SNPs, which could thereby have resulted in reduced power for detecting associations.

Although none of the observed association signals achieved stringent levels of genome wide significance, likely due to the limited sample size of the Indonesian GWAS cohort, the major findings from the study of both Indonesians and Russians does suggest associations at several loci, many of which are located in, or close to immune related genes that have congruous functions toward Th1 axis of the pro-inflammatory IFN-γ activity. IFN-γ is an essential cytokine for the effective control of *M. tuberculosis* in the host, due to its central role in modulating and bridging both the innate and adaptive immunity, impairments in this axis of cytokine activity could render adverse consequences. A previous study conducted on a subset of samples from the same Indonesian cohort, had peripheral blood cells taken from active TB patients, patients undergoing treatment, and healthy controls, and traced for Th1 cytokines production in response to *M. tuberculosis* and mitogen stimulations [12]. The integrity of major pathways involved in Th1 immunity were analyzed, among them IFN-γ level was found to be significantly correlated with TB disease activity and response to curative treatment, that was specific to *M. tuberculosis* stimulation [12]. This change in cytokine activity according to the disease course of pulmonary TB is unlikely due to major defects in IFN-γ itself, since mutations in this molecule and its receptors are known to implicate rare severe infections to otherwise poorly pathogenic mycobacteria [41,42]. Rather, in pulmonary TB, a complex disease with adult onset, it is more likely due to the accumulation of individual subtle effects from variations in genes, such as those suggested from this study that are working together in similar pathways, which might sway the immune responses of the group of susceptible individuals toward active disease.

## Conclusions

Tuberculosis is a complex disease resulting from multiple contributing factors, and the mechanism that triggers active disease is unlikely to be simplistic. Aiming to expand TB disease knowledge, this study took a comprehensive search across the genome, and suggests multiple targets working in novel pathways involved in the host containment of infection with TB, further providing insights on the mechanism of this disease, that could previously be neglected in hypothesis driven approach.

## List of abbreviations

TB: tuberculosis; GWAS: genome wide association scan; SNP: single nucleotide polymorphism; HIV: Human immunodeficiency virus; PC: principal component; MAF: minor allele frequency; HWE: Hardy Weinberg equilibrium; QC: quality control; WHO: World Health Organization; OPA: oligo pool assay; IBS- identity by state; LD- linkage disequilibrium; OR- Odds ratio; QQ plot- quantile-quantile plot; λGC- lambda genomic control inflation factor; CMH: Cochran-Mantel-Haenszel; JPT: HapMap Japanese from Tokyo; CHB: HapMap Han Chinese from Beijing; CEU: HapMap Caucasian from North America.

## Competing interests

The authors declare that they have no competing interests.

## Authors' contributions

All authors contributed in various phases to the writing, and had read and approved the final manuscript. TO and MS were the principal investigators of the study, and supervised it throughout together with RC and MH. BA, ES, RN all played crucial roles in patient and control selection and sampling. EP performed the genotyping, statistical analysis, and the drafting of this manuscript. EV contributed to many discussions and helped writing the manuscript. IA contributed in processing biological samples and managing the database. SM was instrumental in co-designing the project. YB, VN, FD co-ordinate and implemented patient and control selection and sampling sample in Russia. SN participated in sample collection in Russia and association analysis of the Russian data.

## Pre-publication history

The pre-publication history for this paper can be accessed here:

http://www.biomedcentral.com/1471-2350/13/5/prepub

## Supplementary Material

Additional file 1 **Supplementary Figure S1**: Principal component ancestry (PCA) analysis plots of the stage 1 Indonesian GWAS cohort. **Supplementary Figure S2**: Quantile-quantile plot of P value distribution for the association with pulmonary TB in the stage 1 Indonesian GWAS cohort. **Supplementary Figure S3**: Manhattan plot based on P values derived from Trend test association analyses of 95,207 SNPs in 108 PTB cases and 115 controls of stage 1 Indonesian GWAS. Click here for file

Additional file 2 **Supplementary Table S1**: Association results and genotype counts of 251 SNPs (P < 0.05) from the stage 1 and 2 Indonesian study that were carried forward to stage 3 Russian study **Supplementary Table S2**: Association results and genotype counts of nine significant SNPs from the combined meta-analysis results of all three stages. Click here for file
