# Supplementary material for: A genome wide association study of pulmonary tuberculosis susceptibility in Indonesians
Source: BMC Med Genet. 2012 Jan 13;13:5. doi: 10.1186/1471-2350-13-5 (PMC3287960; doi:10.1186/1471-2350-13-5)

Supplementary materials

Supplementary figure 1: Principal component ancestry (PCA) analysis plots of the stage 1 Indonesian GWAS cohort.

Ancestry analysis using principal component (PC) 1 versus 2

A) The ancestry of the Indonesian PTB cases and controls genotyped in stage 1 GWAS, in relative to the international HapMap (Phase1) reference populations. Each dot represents a sample and is color coded according to population groupings.

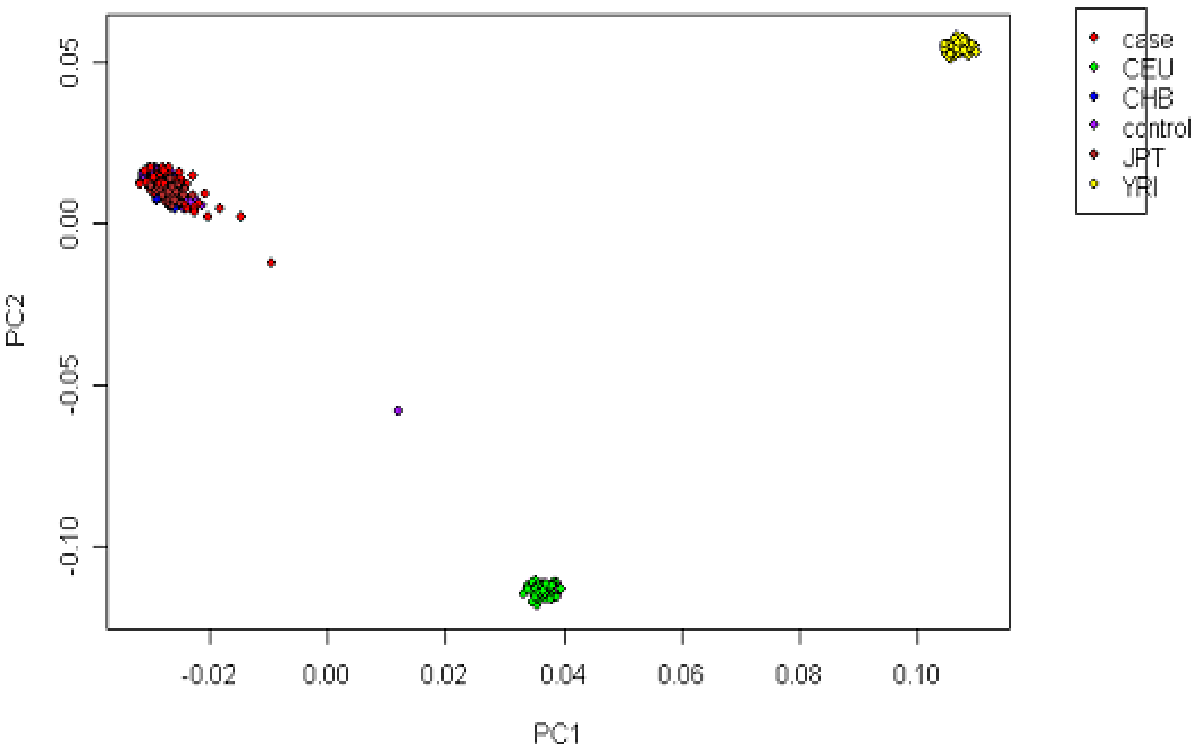

CEU- HapMap Caucasian from North America, CHB- HapMap Han Chinese from Beijing, JPT- HapMap Japanese from Tokyo, YRI- HapMap African from Yoruba

B) The ancestry analysis of the stage 1 PTB cases vs. controls, by case/control status. Each dot represents a sample, cases are color coded in black, while controls are in yellow.

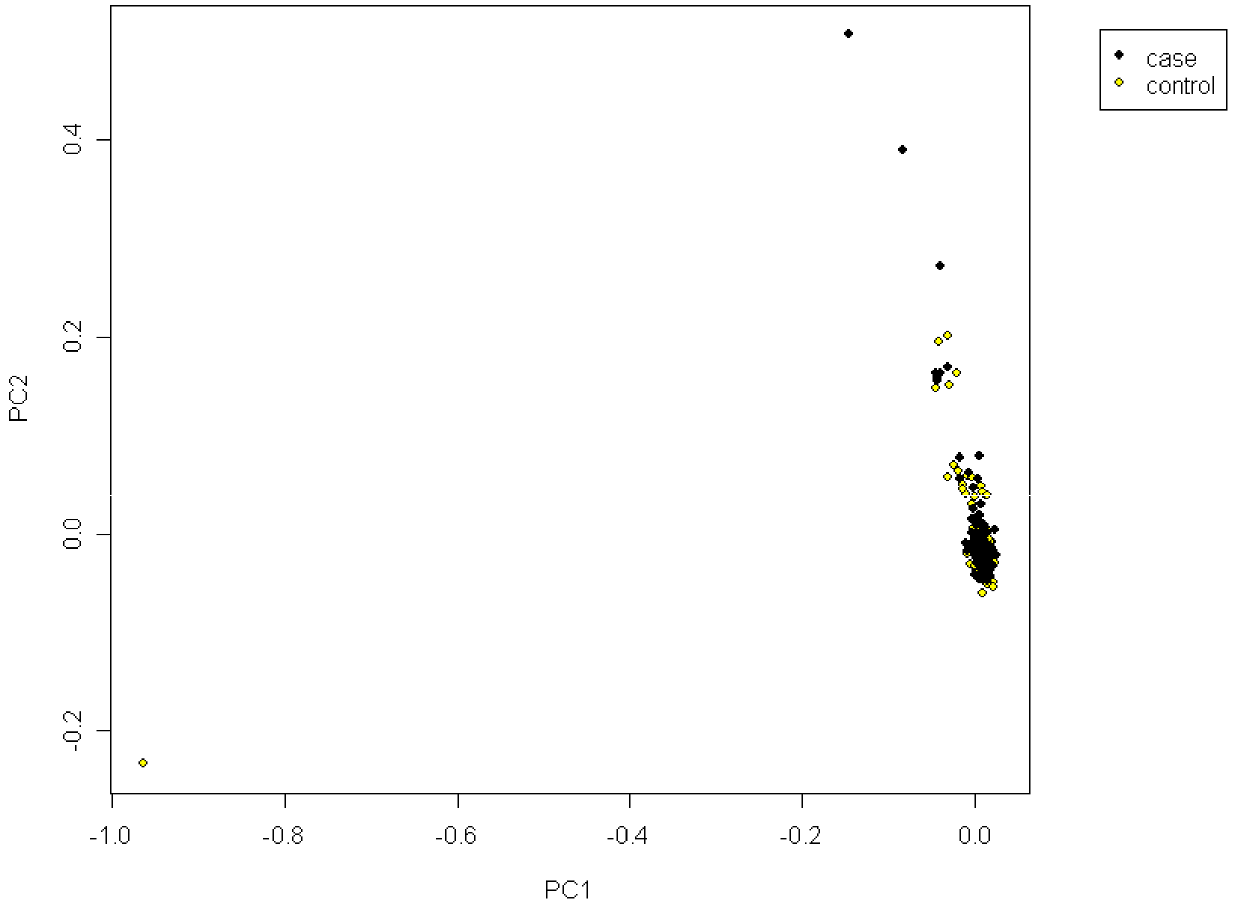

C) The ancestry analysis of the stage 1 PTB cases vs. controls, appended with outliers I.D

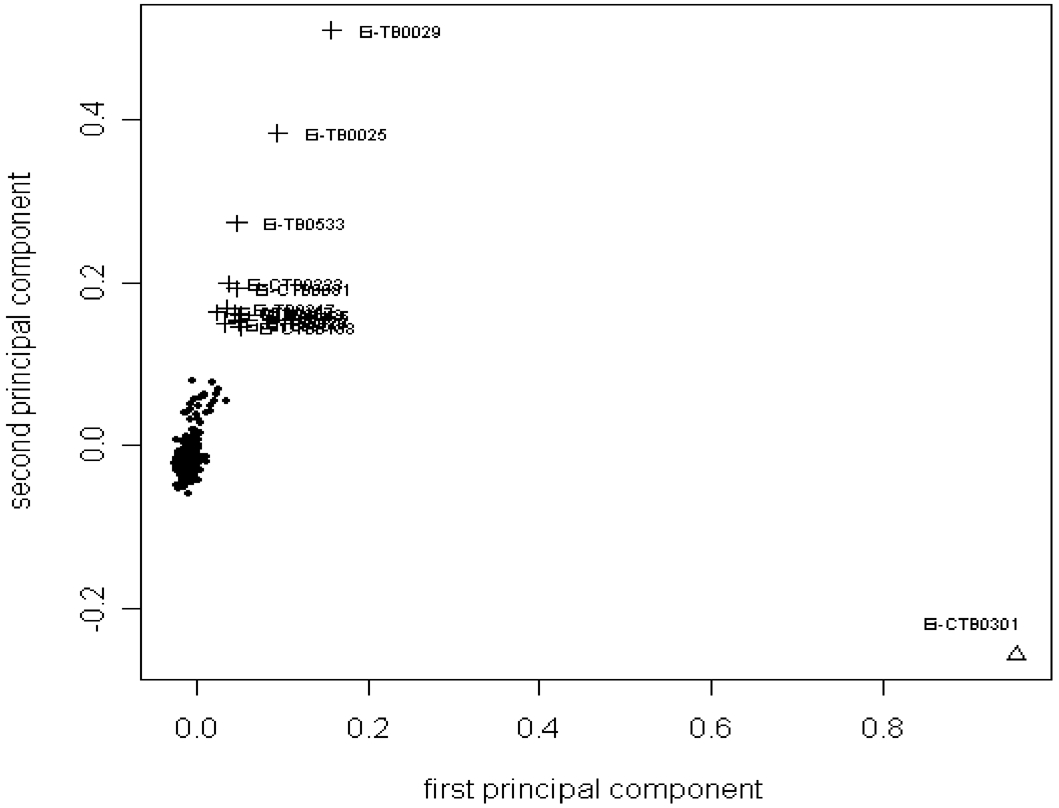

The following outliers were identified based on: PC1 > 0.8 PC2 > 0.2

Sample I.D.	Eigen outliers	Affection status	Ethnicity	Father's ethnicity	Mother's Ethnicity
EITB-0025	yes	case	non-Javanese	Ambon	Ambon
EITB-0029	yes	case	non-Javanese	Aceh	Aceh
EITB-0533	yes	case	non-Javanese	Timor	Kendari
EICTB-0301	yes	control	mixed	India	Jawa

Supplementary figure 2: Quantile-Quantile plot of P value distribution for the association with pulmonary TB in the Stage 1 Indonesian GWAS cohort.
Trend test association analyses of expected versus observed P-values of 95,207 SNPs in 108 PTB cases and 115 controls. Red dots showing deviations from the line of equality indicate either that the theoretical distribution was incorrect, or that the study contains results with value generated by a true association. In this plot, the observed distribution matches the expected distribution closely and shows an excess only in the tail at $P < 10^{-3}$. The median chi-square statistics of the stage 1 genome wide 95,207 loci yield a lambda inflation factor (Devlin and Roeder method) of only 1.003, which indicate that there was no significant population stratification in this study

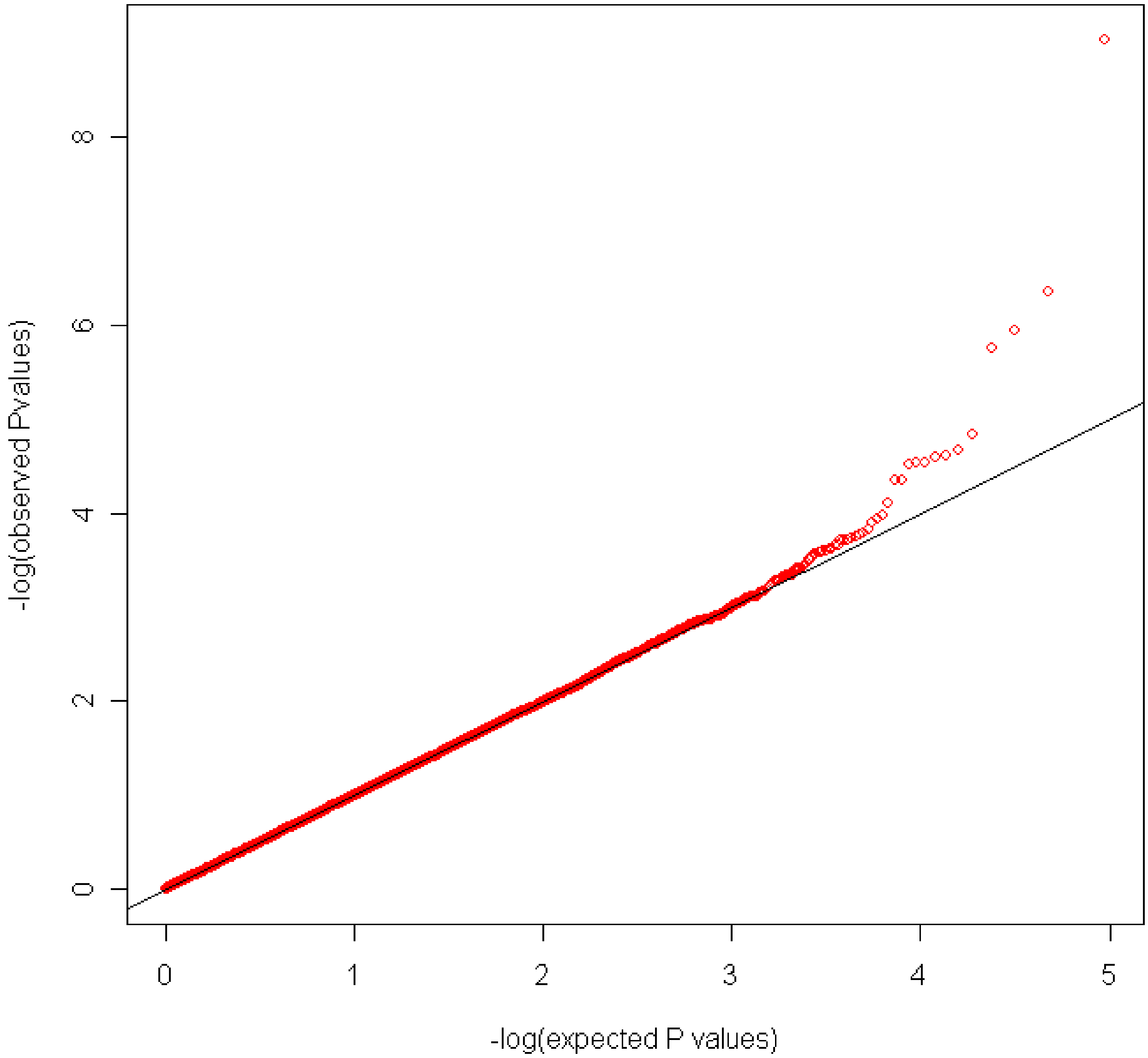

Supplementary figure 3: Manhattan plot based on P values derived from Trend test association analyses of 95,207 SNPs in 108 PTB cases and 115 controls of stage 1 Indonesian GWAS. Each dot represents the SNP –log 10 P value in its color coded chromosomal location.

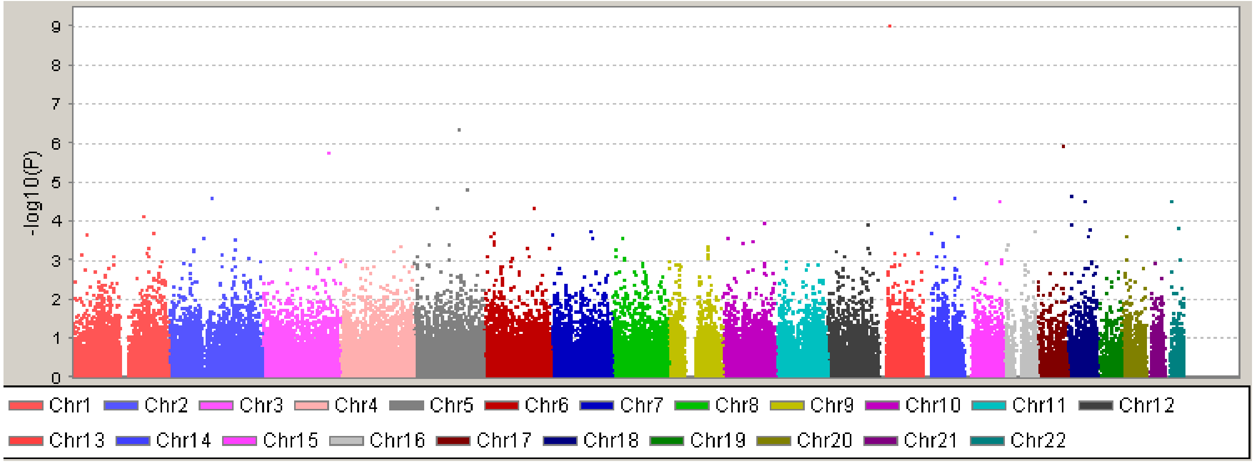

Supplement: Additional file 1 — Supplementary Figure S1: Principal component ancestry (PCA) analysis plots of the stage 1 Indonesian GWAS cohort. Supplementary Figure S2: Quantile-quantile plot of P value distribution for the association with pulmonary TB in the stage 1 Indonesian GWAS cohort. Supplementary Figure S3: Manhattan plot based on P values derived from Trend test association analyses of 95,207 SNPs in 108 PTB cases and 115 controls of stage 1 Indonesian GWAS. [file 1471-2350-13-5-S1.PDF]
